# Association between blood ethylene oxide levels and periodontitis risk: a population-based study

**DOI:** 10.3389/fpubh.2024.1338319

**Published:** 2024-02-07

**Authors:** Yixuan Liu, Nuozhou Liu, Wei Xiong, Ruiyu Wang

**Affiliations:** ^1^State Key Laboratory of Oral Diseases, National Clinical Research Center for Oral Diseases, West China Hospital of Stomatology, Sichuan University, Chengdu, China; ^2^Department of Obstetrics and Gynecology, West China Second University Hospital, Sichuan University, Chengdu, China; ^3^Key Laboratory of Birth Defects and Related Diseases of Women and Children, Sichuan University, Ministry of Education, Chengdu, China; ^4^West China Hospital, West China School of Medicine, Sichuan University, Chengdu, China

**Keywords:** periodontitis, ethylene oxide, NHANES, epidemiology, etiology

## Abstract

**Background:**

The etiopathogenesis of periodontitis is closely associated with environmental conditions. However, the relationship between ethylene oxide exposure and periodontitis risk remains unclear.

**Methods:**

We selected qualified participants from National Health and Nutrition Examination Survey (NHANES) 2013–2014. Periodontitis was identified according to the criteria of the Community Periodontal Index (CPI), Centers for Disease Control and Prevention (CDC)/American Academy of Periodontology (AAP) definition. Ethylene oxide exposure was quantified by hemoglobin adducts of ethylene oxide (HbEO) levels. Log2-transformation was used to normalize HbEO levels. We designed three logistic regression models to explore potential relationship between HbEO and periodontitis. Restricted cubic spline (RCS) and subgroup analysis were also conducted with all covariates adjusted. We performed multivariable linear regression to appraise the association between the risk of periodontitis and different indicators of inflammation, including white blood cells, neutrophils, lymphocytes, and monocytes. Mediation analysis was subsequently performed to examine whether ethylene oxide exposure contributed to periodontitis development through systemic body inflammation.

**Results:**

A total of 1,065 participants aged more than 30 were incorporated in this study. We identified that participants with higher HbEO levels showed increased risk of periodontitis after adjusting for all covariates (OR = 1.49, 95% CI: 1.14, 1.95, *p* = 0.0014). The results of subgroup analysis remained stable. The restricted cubic spline (RCS) curve also revealed a non-linear correlation between log2-transformed HbEO levels with the risk of periodontitis (*p* for nonlinear < 0.001). Mediation analysis indicated that HbEO level was significantly associated with four inflammatory mediators, with the mediated proportions of 14.44% (*p* < 0.001) for white blood cell, 9.62% (*p* < 0.001) for neutrophil, 6.17% (*p* = 0.006) for lymphocyte, and 6.72% (*p* < 0.001) for monocyte.

**Conclusion:**

Participants with higher ethylene oxide exposure showed higher risk of periodontitis, which was partially mediated by systemic body inflammation. More well-designed longitudinal studies should be carried out to validate this relationship.

## 1 Introduction

Periodontitis is a chronic inflammatory disease characterized by impaired integrity of tooth-supporting tissue, which eventually leads to tooth looseness and the loss of teeth if not properly treated. The high prevalence of periodontitis severely affects patients’ life quality and causes enormous socioeconomic burden (1, 2). The etiology of periodontitis is very complex, including but not restricted to environment, life style, diet, and genetic susceptibility (3). Multiple environmental risk factors were associated periodontitis, particularly smoking and particulate matter exposure. These risk factors were considered to have significant pro-inflammatory effects, which may lead to systemic inflammatory reaction and might contribute to periodontitis development (4, 5). Unlike genetic risk factor for periodontitis, environmental risk factors are considered modifiable, and identifying potential environment-related risk is critical to periodontitis management (6).

Ethylene oxide is a common environmental organic compound derived from the metabolism of ethylene. Hemoglobin adducts of ethylene oxide (HbEO) is a significantly sensitive biomarker for ethylene oxide assessment because of its longer half-life *in vivo*. Ethylene oxide has been widely applied as intermediates for various compounds, including ethylene glycols, glycol ethers, and other ethoxylated products (7). In addition, ethylene oxide is an important sterilizing agent for oral medical devices with excellent bactericidal, sporicidal, and virucidal activity (8). Since individuals can be exposed to ethylene oxide through inhalation, it is also recognized as an environmental pollutant derived from tobacco smoke and industrial process. Previous studies indicated that, as a highly reactive volatile organic compound, people exposed to excessive ethylene oxide were more likely to have a higher risk of cardiovascular diseases, respiratory diseases, and cancer (9–11).

However, the relevance of ethylene oxide exposure with periodontitis development remained unclear. Increasing evidence showed that ethylene oxide exposure could intensify systemic body inflammation that affected the development of periodontitis (12, 13). On the one hand, the inflammatory response is a kind of defense mechanism against the invasion of external pathogens. On the other hand, an improperly controlled inflammatory response can cause irreversible damage to periodontal tissues with typical signs of periodontitis such as deep periodontal pockets, attachment loss, and even tooth loss (14). Uncontrolled systemic inflammation not only contributes to the development of periodontitis but also its comorbidities, like cardiovascular and respiratory diseases (3, 15). Since periodontitis is also a chronic systemic inflammatory disease, we hypothesized that a correlation exists between ethylene oxide exposure and risk of periodontitis possibly mediated by systemic body inflammation.

Here, our study aimed to explore the hypothesis that ethylene oxide exposure might contribute to periodontitis, which partially mediated by systemic inflammation, using statistics from the National Health and Nutrition Examination Survey (NHANES) 2013–2014.

## 2 Materials and methods

### 2.1 Study design and population

The datasets utilized in our study were based on the National Health and Nutrition Examination Survey (NHANES) 2013–2014, a cross-sectional survey conducted by the National Center for Health Statistics (NCHS). NHANES was used to investigate the health and nutritional status of noninstitutionalized US individuals with a stratified multistage representative sample. All the participants’ data collection can be publicly obtained at www.cdc.gov/nchs/nhanes.htm. The original NHANES 2013–2014 dataset was carried out in US populations with approvement from the Centers for Disease Control (CDC) and Prevention National Increase for Health Statistics Research (NCHS) Ethics Review Board. All the participants included have provided written informed consent, which can be accessed from https://www.cdc.gov/nchs/nhanes/irba98.htm. This paper followed the Strengthening the Reporting of Observational Studies in Epidemiology (STROBE) guideline (16).

**Figure 1:**
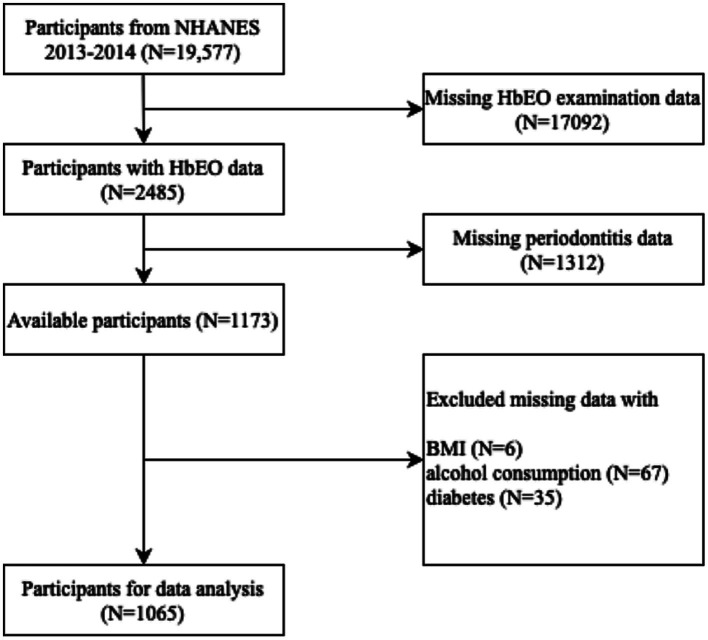
Flow chart of process for selection and inclusion of participants. NHANES, National Health and Nutrition Examination Survey; HbEO, hemoglobin adducts of ethylene.

A total of 19,577 participants were included from 2013 to 2014 cycles in NHANES. Incomplete data of household interviews and physical examinations were excluded (*n* = 18,512). As a result, 1,065 participants aged 30 or older were enrolled for the data analysis (Figure 1).

### 2.2 Assessment of periodontitis

Participants aged ≥ 30 were included for a full-mouth periodontal probing examinations conducted by calibrated examiners.^1^ All periodontitis cases reached the criteria of the Community Periodontal Index (CPI), Centers for Disease Control and Prevention (CDC)/American Academy of Periodontology (AAP) definition. The grade of periodontal status was diagnosed according to the CDC/AAP definitions (17). The severity of periodontitis can be categorized as three levels (Supplementary Table 1). Participants were defined as periodontitis cases if they met the criteria of either mild, moderate, or severe periodontitis, while the rest of them were defined as non-periodontitis.

### 2.3 Assessment of blood ethylene oxide

We exploited a series of standard control strategies to find valid IVs that satisfied three, the reaction product of ethylene oxide with hemoglobin, was utilized to quantify cumulative ethylene oxide exposure for the past 4 months (18, 19). Hemoglobin adducts of ethylene oxide has been testified as a significantly sensitive mark for ethylene oxide exposure because of its longer half-life *in vivo*. Washed-packed blood samples supplied by participants in the morning were processed and stored under −30°C conditions until shipped to the National Center for Environmental Health for evaluation. The modified Edman reaction by high-performance liquid chromatography coupled with tandem mass spectrometry (HPLC-MS/MS) was utilized to assess HbEO in human whole blood or erythrocytes, using the reaction products with the N-terminal valine residue of the hemoglobin protein chains (N-[2-carbamoyl ethyl] valine and N-[2-hydroxycarbamoyl-ethyl] valine ethylene oxide adducts) measured. The results of measurements were exhibited as pmol/g Hb. The accuracy of the test results conformed the quality control and quality assurance performance standards of the NCEH Laboratory Sciences Division. More details of the measurement are available at the NHANES Laboratory/Medical Technologist Procedures Manual.^2^

### 2.4 Covariates

Additional covariates related to periodontitis were comprehensively incorporated in our study, including: (1) demographic characteristics: age (<50, 50~70, and ≥70), gender (male and female), ethnicity (Mexican American, other Hispanic, non-Hispanic white, non-Hispanic black, and other race including multi-racial), alcohol consumption (<12 alcohol drinks/year and ≥12 alcohol drinks/year), smoking (<100 cigarettes in life and ≥100 cigarettes in life). (2) physical examinations parameters: BMI (<25 kg/m^2^ and ≥25 kg/m^2^). (3) medical conditions: diabetes (yes and no). These 7 cofounding factors have all been identified as risk factors of periodontitis (20–23).

Alcohol users were defined as participants who consumed at least 12 alcohol drinks in a single calendar year. Smokers were defined as individuals who had lifetime use of ≥100 cigarettes. BMI was calculated by dividing the weight (kg) by the square of height in meters (m^2^). The diabetes status was identified according to previous self-reported. Individuals who answered “yes” to the question “Have you ever been told by a doctor or other health professional that you had diabetes?” were confirmed the presence of diabetes.

### 2.5 Statistical analysis

Given to the elaborate sampling design of NHANES, we implemented sample weighting, clustering, and stratification during statistical analysis process. R package “survey” with command “svydesign” was utilized to consider stratified multistage representative sample settings of NHANES (24). Kolmogorov–Smirnov statistical test was conducted in advance to detect the normal distribution of continuous variables. Categorical variables were analyzed by chi-square tests and presented as proportions (%). Continuous variables were presented as the mean ± standard deviation (SD) with normal distribution or medians (IQRs) with non-normal distribution. For normally distributed continuous variables, student t-test was applied to examine the difference, while Mann–Whitney U-test for non-normally distributed variables. Log2-transformed HbEO levels were divided into four intervals in accordance with quartiles and multiple logistic regression models were performed to estimate odds ratios (OR) and 95% confidence intervals (95% CI). In addition, we designed three logistic regression models to assess potential relationship between HbEO and periodontitis. Model 1 was a crude model with no covariates adjusted. Model 2 was adjusted for age, gender and ethnicity. Model 3 was adjusted for all covariates, including age, gender, ethnicity, alcohol consumption, smoking, BMI, and diabetes. Base on this extended model, we carried out restricted cubic spline (RCS) with three knots for dose–response analysis. Subgroup analysis was conducted according to age, gender, ethnicity, alcohol consumption, smoking, BMI and diabetes, as the same way in Model 3. Moreover, we performed multivariable linear regression to appraise the association between the risk of periodontitis and different indicators of inflammation, including white blood cells, neutrophils, Lymphocytes, and monocytes. Mediation analysis was subsequently performed to examine whether ethylene oxide exposure contributed to periodontitis development through systemic body inflammation. All data analysis was operated in R (version 4.1.3) and Python. Two-side *p* < 0.05 was regarded as statistically significant.

## 3 Results

### 3.1 Baseline characteristics

**Figure 2:**
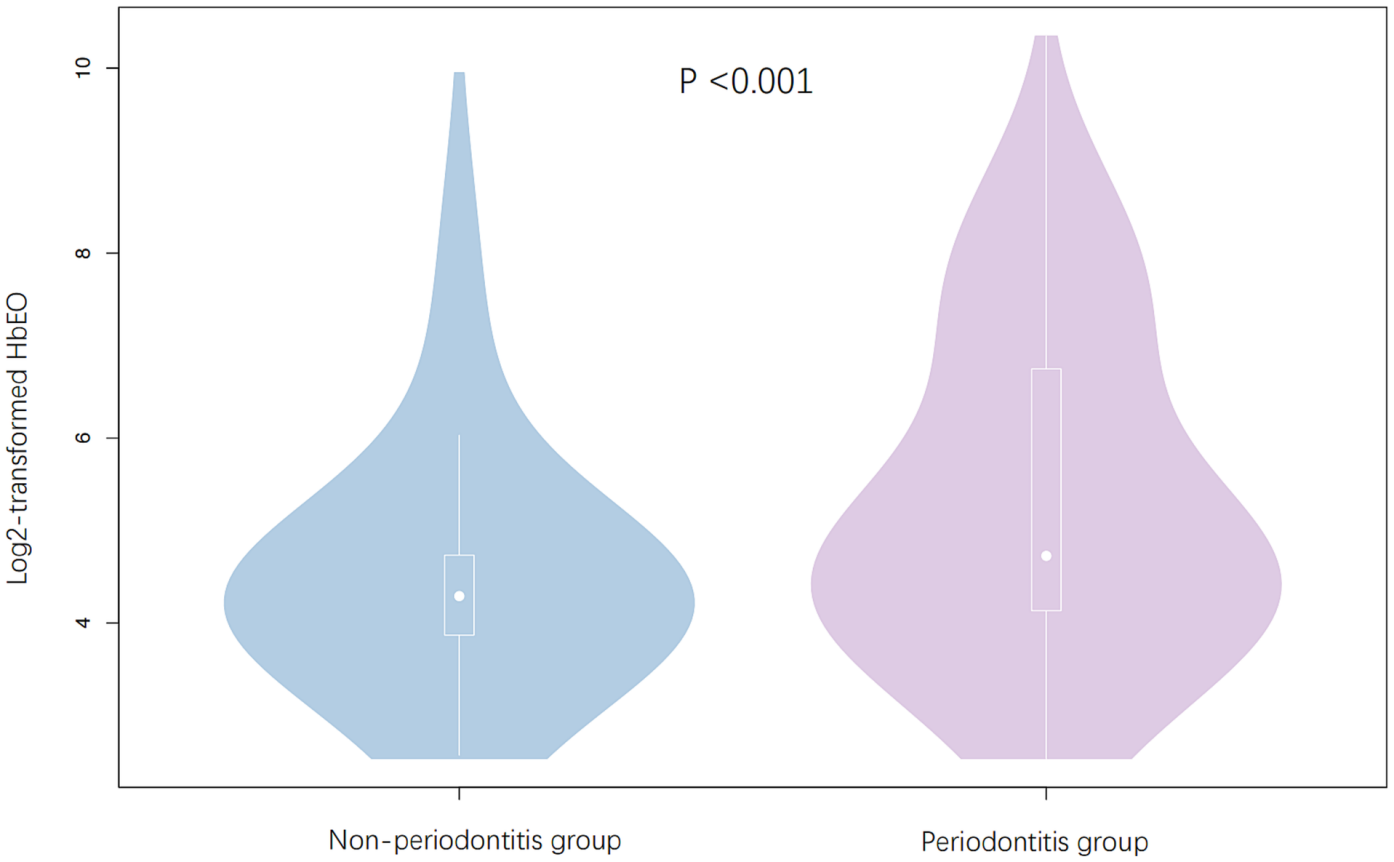
Log2-transformed HbEO levels in non-periodontitis group and periodontitis group.

We enrolled 1,065 appropriate participants from NHANES 2013–2014 cycle for data analysis. As demonstrated in Figure 2, periodontitis group has a significantly higher log2-transformed HbEO levels than non-periodontitis group (*p* < 0.001).

More details about baseline characteristics are presented in Table 1. Overall, 502 (47.1%) participants were diagnosed as periodontitis. Participants with periodontitis were more likely to be older, male (59.96%), non-Hispanic black (40.24%), and smokers (55.58%). While no significant difference was observed in alcohol consumption (*p* = 0.1691), BMI (*p* = 0.5946), or diabetes (*p* = 0.1221).

**Table 1:** Characteristics of participants based on PD status.

Variables	Non-PD group (*N* = 563)	PD group (*N* = 502)	Value of *p*
Age (years)			<0.0001
(median [IQR])	47.000 [38.000, 59.000]	55.000 [43.000, 65.000]	
Gender, *n* (%)			<0.0001
Male	230 (40.85)	301 (59.96)	
Female	333 (59.15)	201 (40.04)	
Ethnicity, *n* (%)			0.0255
Mexican American	73 (12.97)	82 (16.33)	
Other Hispanic	48 (8.53)	42 (8.37)	
Non-Hispanic white	261 (46.36)	202 (40.24)	
Non-Hispanic black	94 (16.70)	114 (22.71)	
Other race including multi-racial	87 (15.45)	62 (12.35)	
Alcohol consumption, *n* (%)			0.1691
<12 alcohol drinks/year	160 (28.42)	123 (24.50)	
≥12 alcohol drinks/year	403 (71.58)	379 (75.50)	
Smoking, *n* (%)			<0.0001
<100 cigarettes in life	364 (64.65)	223 (44.42)	
≥100 cigarettes in life	199 (35.35)	279 (55.58)	
BMI (kg/m^2^)			0.5946
<25.0	155 (27.53)	130 (25.90)	
≥25.0	408 (72.47)	372 (74.10)	
Diabetes, *n* (%)			0.1221
Yes	65 (11.55)	75 (14.94)	
No	498 (88.45)	427 (85.06)	

BMI, body mass index.

### 3.2 Association between HbEO and periodontitis

The association between HbEO and periodontitis is presented in Table 2. We carried out univariate logistic regression analysis to investigate overall association between continuous log2-transformed HbEO and the prevalence of periodontitis, with a notable difference detected in crude model 1(OR = 1.49, 95% CI = 1.28–1.73, *p* < 0.001). This association remained stable after adjusting for covariates in both model 2 (OR = 1.57, 95% CI = 1.28–1.92, *p* < 0.001) and model 3 (OR = 1.49, 95% CI = 1.14–1.95, *p* = 0.014) by multivariate logistic regression analysis.

**Table 2:** Multivariate logistic regression analysis of log2-transformed HbEO for risk of PD.

	Model 1		Model 2		Model 3	
	Crude OR (95%CI)	*p*-value	Adjusted OR (95%CI)	*p*-value	Adjusted OR (95%CI)	*p*-value
Continuous log2-HbEO	1.49 (1.28–1.73)	<0.001	1.57 (1.28–1.92)	<0.001	1.49 (1.14–1.95)	0.014
Q1 group	Reference		Reference		Reference	
Q2 group	1.97 (1.05–3.69)	0.037	1.85(0.87–3.96)	0.095	1.72 (0.42–7.11)	0.241
Q3 group	5.43 (2.76–10.72)	<0.001	7.71 (3.21–18.51)	0.001	6.32 (1.29–30.94)	0.038
Q4 group	4.18(1.83–9.58)	0.003	5.18 (1.65–16.22)	0.012	4.30 (0.50–36.89)	0.010
*P* for trend	<0.001		0.030		0.014	

Model 1 was a crude model with no covariates adjusted. Model 2 was adjusted for age, gender, and ethnicity. Model 3 was adjusted for all covariates. HbEO, hemoglobin adducts of ethylene oxide; OR, odd ratio; CI, confidence interval.

Compared with Q1 group for reference, Q4 group indicated a higher risk of periodontitis in all three models: model 1 (OR = 4.18, 95% CI = 1.83–9.58, *p* = 0.003, *P* for trend < 0.001), model 2 (OR = 5.18, 95% CI = 1.65–16.22, *p* = 0.012, *P* for trend = 0.030), and model 3 (OR = 4.30, 95% CI = 0.50–36.89, *p* = 0.010, *P* for trend = 0.014).

**Figure 3:**
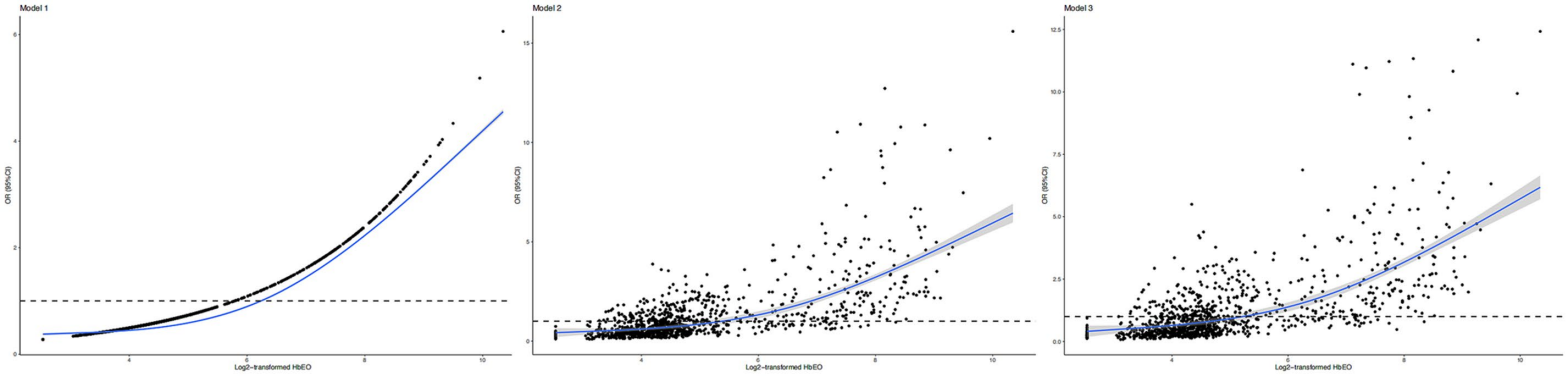
Restricted cubic spline (RCS) plots of the association of HbEO levels with periodontitis. **(A)** Model 1: no covariates adjusted; **(B)** Model 2: adjusted for age, gender, and ethnicity. **(C)** Model 3: adjusted for all covariates. OR, odd ratio; (CI), confidence interval; HbEO, hemoglobin adducts of ethylene oxide.

The restricted cubic spline (RCS) curve also revealed a positive nonlinear correlation of log2-transformed HbEO levels with the risk of periodontitis in both adjusted and unadjusted model (Figure 3; *p* for non-linearity < 0.001). Briefly, higher HbEO levels were associated with an increased risk of periodontitis.

### 3.3 Subgroup analysis

As shown in Table 3, no significant interaction was identified (all *p* for interaction > 0.05) in all subgroups. The influence of HbEO on periodontitis was generally consistent among different age, gender, alcohol consumption, smoking, BMI, and diabetes subgroups. Notably, the association between ethylene oxide and periodontitis was non-significant among participants aged ≥70 (OR = 1.75, 95% CI = 0.53–5.75) or smoked ≥ 100 cigarettes in life (OR = 1.09, 95% CI = 0.65–1.82).

**Table 3:** Subgroup analysis of the association of HbEO levels with PD.

Variables	OR	95% CI	*p* for interaction
Age			0.6611
30–49 years	1.49	1.01–2.18	
50–69 years	1.35	1.01–1.82	
≥70 years	1.75	0.53–5.75	
Gender			0.6859
Male	1.55	1.02–2.35	
Female	1.47	1.16–1.86	
Alcohol consumption			0.6569
<12 alcohol drinks/year	1.87	1.14–3.08	
≥12 alcohol drinks/year	1.46	1.12–1.91	
Smoking			0.2691
<100 cigarettes in life	1.57	1.17–2.10	
≥100 cigarettes in life	1.09	0.65–1.82	
BMI			0.9291
<25.0	1.46	1.18–1.82	
≥25.0	1.52	1.11–2.09	
Diabetes			0.6021
Yes	1.42	1.10–1.83	
No	1.64	1.03–2.61	

HbEO, hemoglobin adducts of ethylene oxide; OR, odds ratio; CI, confidence interval; BMI, body mass index.

### 3.4 Mediation analysis

Multiple linear regression analysis demonstrated that there were significant correlations between log2-transformed HbEO and white blood cells (*β* = 0.34, 95% CI = 0.25–0.43, *p* < 0.001, SE = 0.05), neutrophils (*β* = 0.22, 95% CI = 0.15–0.30, *p* < 0.001, SE = 0.04), lymphocyte (*β* = 0.09, 95% CI = 0.06–0.12, *p* < 0.001, SE = 0.02), and monocyte (*β* = 0.02, 95% CI = 0.008–0.025, *p* < 0.001, SE = 0.004; Table 4).

**Table 4:** Multiple linear regression of log2-transformed HbEO with inflammatory indicators.

Mediators	*β*	95% CI	*p*-value	SE
White blood cell count	0.34	0.25–0.43	<0.001	0.05
Neutrophil count	0.22	0.15–0.30	<0.001	0.04
Lymphocyte count	0.09	0.06–0.12	<0.001	0.02
Monocyte count	0.02	0.008–0.025	<0.001	0.004

This model was adjusted for age, gender, ethnicity, alcohol consumption, smoking, BMI, and diabetes. HbEO, hemoglobin adducts of ethylene oxide; CI, confidence interval; SE, standard error.

In addition, mediation analysis identified a mediation proportion of 14.44% (*p* < 0.001) for white blood cells (Supplementary Figure S1), 9.62% (*p* < 0.001) for neutrophils (Supplementary Figure S2), 6.17% (*p* = 0.006) for lymphocyte (Supplementary Figure S3), and 6.72% (*p* < 0.001) for monocyte (Supplementary Figure S4).

## 4 Discussion

To our knowledge, this is the first large-scale cross-sectional study investigating the association between environmental ethylene oxide exposure and periodontitis risk among adults in the United States and the mediation effects of systemic inflammation (including white blood cell count, neutrophil count, lymphocyte count, and monocyte count). We identified that participants with higher HbEO showed higher risk of periodontitis, which was partially mediated by systemic inflammation (Table 5).

**Table 5:** The mediation effects of inflammatory indicators on the association between log2-transformed HbEO and PD.

Mediators	Total effects	Indirect effects	Direct effects	Mediated proportion (%)	*p*-value
	*β* (95% CI)	*β* (95% CI)	*β* (95% CI)		
White blood cell count	0.05 (0.04,0.050)	0.01 (0.003,0.01)	0.04 (0.03–0.05)	14.44	<0.001
Neutrophil count	0.05 (0.04,0.05)	0.005 (0.002,0.01)	0.04 (0.04,0.05)	9.62	<0.001
Lymphocyte count	0.05 (0.04,0.05)	0.003 (0.001,0.01)	0.04 (0.04,0.05)	6.17	0.006
Monocyte count	0.05 (0.04,0.05)	0.003 (0.001,0.01)	0.04 (0.04,0.05)	6.72	<0.001

This model was adjusted for age, gender, ethnicity, alcohol consumption, smoking, BMI, and diabetes. HbEO, hemoglobin adducts of ethylene oxide; CI, confidence interval.

It is well-established that periodontitis is a systemic inflammatory disease with complex etiologies at multiple levels, including environmental pollutant exposure, genetics, dysbiotic microbe infection and life styles (3, 25, 26). The prevalence rate of periodontitis in this study was 47.1%, which was generally in line with epidemiological trend report in US but lower than the pool estimate rate of 62% reported by a recent meta-analysis (27, 28). The prevalence rate difference might derive from different population settings and diagnostic criteria and cofounded by the age of participants. Notably, the application of full-mouth periodontal examination combined with details on demographic information and medical conditions among NHANES participants aged more than 30 provided a relatively more precise estimate for periodontitis prevalence (27). Since environmental risk factor like cigarette smoking is considered as one of the most important modifiable risk factors for periodontitis prevention and treatment, identifying potential environmental risk factors is critical to periodontitis management (29). Previous studies have shown that exposure to ethylene oxide have mutagenic and genotoxic effects and can produce numerous unfavorable health impacts (30–32). Given the potential mutagenic and genotoxic effects of ethylene oxide, it has been long hypothesized that ethylene oxide exposure from both skin and respiratory tract can increase the risk of malignancies (33–35). A recent cohort study based on the US Environmental Protection Agency’s Toxics Release Inventory found that participants locating within 10 km from EtO-emitting sites showed increased risk of *in situ* breast cancer but not invasive breast cancer or non-Hodgkin lymphoma (10). And occupational exposure to ethylene oxide might increase mortality risk from lymphatic and hematopoietic malignancies (36). However, the relationship between ethylene oxide exposure and risk of malignancies remains controversial. A recent systematic review assessing the potential carcinogenicity of ethylene oxide exposure from respiratory tract suggested that there was no association between ethylene oxide exposure and breast cancer, stomach cancer, and lymphohematopoietic malignancies (31). Mundt et al. stated that there was only limited evidence supporting a causal association between ethylene oxide exposure and risk of malignancies (37). As for non-malignant diseases, He et al. reported that people with higher HbEO showed an increased risk of chronic obstructive pulmonary disease (COperiodontitis) partially mediated by inflammation (11). The prevalence rates of hypertension and high diastolic blood pressure were also significantly higher among people with higher HbEO level (38). Elevated level of HbEO was also associated with higher HbA1c, lower high-density lipoprotein cholesterol, and higher risk of diabetes mellitus (39). Peng et al. also reported a dose-dependent risk of kidney stones among people exposed to ethylene oxide (40). A significantly increased risk of spontaneous abortion and pregnancy loss was associated with ethylene oxide exposure during pregnancy. However, there is no existing study concentrating on the relationship between ethylene oxide exposure and periodontitis among general population. The current study found that people with higher HbEO level had significantly increased risk of periodontitis.

The underlying mechanism linking ethylene oxide exposure to incident periodontitis are still unclear. Our results firstly demonstrated that systemic inflammation could contribute to periodontitis development when people exposing to ethylene oxide based on epidemiological analysis, which was generally in line with previous researches. Inflammation was considered as a core part of periodontitis pathogenesis for a long period of time (41–43). Periodontitis patients always showed an obvious systemic inflammatory condition with increased level of white blood cells, segmented neutrophils, and inflammatory cytokines (44, 45). Both innate and adaptive immune response are involved in host–pathogen interactions and produce systemic pro-inflammatory milieu with elevated levels of interleukins, interferon-γ, tumor necrosis factor, and antibodies against microbial biofilm in dental plate (25). Furthermore, this host-pathogen interaction could impair periodontal epithelium leading to systemic periodontal pathogen invasion and produce harmful consequences (25, 46). Mendes et al. reported that diet-induced inflammation was associated with higher risk of periodontitis, which was partially mediated by systemic body inflammation (47). Previous studies have also indicated a significant association between ethylene oxide exposure and inflammation. Lynch et al. firstly discovered that long-period ethylene oxide exposure through respiratory tract cause inflammatory lesions in F344 rats (11, 13). Short-term repeated inhalation of ethylene oxide produced inflammatory response in rats and caused moderate to severe alveolitis after 5-day exposure (48). Sterilization procedures using ethylene oxide has also been suspected for producing post-operative inflammatory for many years (49–51). Li et al. found that ethylene oxide exposure was closely linked with unfavorable serum lipid profiles, with systemic inflammation as a key mediator (18). ethylene oxide exposure might increase the risk of asthma in general population similarly mediating by systemic inflammation (52).

This study possesses multiple strengths. Firstly, this is the first large-scale cross-sectional study assessing the association between HbEO and periodontitis risk among United States residents from NHANES. A subsequent mediation analysis was also conducted. Important cofounders for periodontitis like smoking, alcohol consumption and diabetes were adjusted. Sample weights for NHANES were carefully considered, and the STROBE guideline was followed when reporting our results. Lastly, ethylene oxide has become the mostly preferred sterilization method for medical devices because of its effective bactericidal, sporicidal, and virucidal activity (8). And the sharp increases in the demand for personal protective equipment (PPE) during COVID-19 pandemic may also increase the chance of ethylene oxide exposure. Unlike individual genetic susceptibility for periodontitis, environmental risk factors are considered comparatively modifiable, thus residue control of ethylene oxide is required and practical for periodontitis management.

However, this study still had some limitations. Firstly, the cross-sectional study design hindered us to make causal inference between HbEO and risk of periodontitis. Although NHANES analytical protocol recommended combine different cycles to recruit more participants and improve the stability of data estimates, we only select NHANES 2013–2014 because only this cycle documented full information on both HbEO and periodontitis (53). Although the association between ethylene oxide exposure and periodontitis could be affected by other environmental pollutant exposure, such as heavy metals and multiple polyaromatic hydrocarbons (54, 55), we could not consider these above due to limited participant number. And we did not classify the severity of periodontitis in our statistical analysis due to limited number of participants. To be noted, since only ethylene oxide levels for those age ≥ 30 was documented in NHANES, we could not incorporate age groups ≤ 30 into statistical analysis. The definition of smoking and alcohol consumption was solely based on personal interview, where recall bias was inevitable. Although HbEO was considered as a cumulative indicator for ethylene oxide exposure for at least 4 months, it would be better if ethylene oxide exposure was measured dynamically (18, 19). Lastly, we could not avoid residual confounding because of the complex etiopathogenesis of periodontitis.

## 5 Conclusion

Participants with higher ethylene oxide exposure showed higher risk of periodontitis, which was partially mediated by systemic body inflammation. More well-designed longitudinal studies should be carried out to validate this relationship.

### Resource identification initiative

1. NHANES, RRID:SCR_013201.
2. R Project for Statistical Computing, RRID:SCR_001905.

## Data availability statement

The datasets presented in this study can be found in online repositories. The names of the repository/repositories and accession number(s) can be found in the article/Supplementary material.

## Ethics statement

The studies involving humans were approved by the Centers for Disease Control (CDC) and Prevention National Increase for Health Statistics Research (NCHS) Ethics Review Board. The studies were conducted in accordance with the local legislation and institutional requirements. The participants provided their written informed consent to participate in this study.

## Author contributions

YL: Conceptualization, Data curation, Formal analysis, Investigation, Methodology, Resources, Software, Validation, Visualization, Writing – original draft. NL: Conceptualization, Data curation, Formal analysis, Investigation, Methodology, Resources, Software, Validation, Visualization, Writing – original draft. WX: Formal analysis, Investigation, Resources, Validation, Writing – original draft. RW: Funding acquisition, Supervision, Writing – review & editing.

## Footnotes

^1^ https://doi.org/10.1111/jphd.12056

^2^ https://wwwn.cdc.gov/Nchs/Nhanes/2013-2014/ETHOX_H.htm
