# Supplementary material for: Association between blood ethylene oxide levels and periodontitis risk: a population-based study
Source: Front Public Health. 2024 Feb 7;12:1338319. doi: 10.3389/fpubh.2024.1338319 (PMC10879552; doi:10.3389/fpubh.2024.1338319)
Supplement: Supplementary file 1 [file Table_1.DOCX]

Supplementary Material

# Supplementary Figures and Tables

## Supplementary Figures

Figure S1. The mediation effects of white blood cell count on the association between log2-transformed HbEO and PD.

Figure S2. The mediation effects of neutrophil count on the association between log2-transformed HbEO and PD.

Figure S3. The mediation effects of lymphocyte count on the association between log2-transformed HbEO and PD.

Figure S4. The mediation effects of monocyte count on the association between log2-transformed HbEO and PD.

## Supplementary Tables

Table S1. Periodontitis classification criteria according to the probing depth (PD) and clinical attachment level (AL).

| Classification | Criteria |
| --- | --- |
| No periodontitis | No evidence of mild, moderate, or severe PD |
| Mild periodontitis | ≥2 interproximal sites with AL ≥3 mm, and ≥2 interproximal sites with PD ≥4 mm (not on the same tooth) or one site with PD ≥5 mm |
| Moderate periodontitis | ≥2 interproximal sites with AL ≥4 mm (not on the same tooth), or ≥2 interproximal sites with PD ≥5 mm (not on the same tooth) |
| Severe periodontitis | ≥2 interproximal sites with AL ≥6 mm (not on the same tooth) and ≥1 interproximal site with PD ≥5 mm |
